# Efficacy and safety of talazoparib in Japanese patients with germline *BRCA-*mutated locally advanced or metastatic breast cancer: results of the phase 1 dose-expansion study

**DOI:** 10.1007/s12282-022-01390-w

**Published:** 2022-07-30

**Authors:** Haruru Kotani, Norikazu Masuda, Toshinari Yamashita, Yoichi Naito, Tetsuhiko Taira, Kenichi Inoue, Masato Takahashi, Kan Yonemori, Shigeyuki Toyoizumi, Yuko Mori, Takashi Nagasawa, Natsuki Hori, Hiroji Iwata

**Affiliations:** 1 Aichi Cancer Center, 1-1 Kanokoden, Chikusa-ku, Nagoya, 464-8681 Japan; 2 National Hospital Organization Osaka National Hospital, 2-1-14 Hoenzaka, Chuo-ku, Osaka-shi, Osaka 540-0006 Japan; 3 Kanagawa Cancer Center, 2-3-2 Nakao, Asahi-ku, Yokohama, Kanagawa 241-8515 Japan; 4 National Cancer Center Hospital East, 6-5-1, Kashiwanoha, Kashiwa, Chiba 277-8577 Japan; 5 Hakuaikai Medical Corporation Sagara Hospital, 3-31 Matsubara-cho Kagoshima, Kagoshima, 892-0833 Japan; 6 Saitama Cancer Center, 780 Komuro, Ina-machi, Kitaadachi-gun, Saitama, 362-0806 Japan; 7 National Hospital Organization Hokkaido Cancer Center, Kikusui 4-jo 2-chome 3-54 Shiroishi-ku, Sapporo, Hokkaido 003-0804 Japan; 8 National Cancer Center Hospital, 5-1-1 Tsukiji, Chuo-ku, Tokyo, 104-0045 Japan; 9 Pfizer R&D Japan, Shinjuku Bunka Quint Bldg. 3-22-7, Yoyogi, Shibuya-ku, Tokyo, 151-0053 Japan; 10 Present Address: Department of Breast and Endocrine Surgery, Nagoya University Graduate School of Medicine, 65 Tsurumai-cho, Showa-ku, Nagoya, 466-8550 Japan

**Keywords:** Talazoparib, PARP inhibitor, Japanese patients, *BRCA*, Breast cancer

## Abstract

**Background:**

Talazoparib, a poly(ADP-ribose) polymerase enzyme inhibitor, is approved for the treatment of patients with germline *BRCA1/2* (g*BRCA1/2*)-mutated HER2-negative advanced breast cancer. This two-part study, a recently published dose-escalation part followed by the dose-expansion part reported here, evaluated the efficacy and safety of talazoparib in Japanese patients with g*BRCA1/2*-mutated advanced breast cancer.

**Methods:**

In this open-label, multicenter phase 1 study (NCT03343054), the primary endpoint of the dose-expansion part was confirmed objective response rate (ORR), determined by investigator assessment (RECIST 1.1). Secondary endpoints included progression-free survival (PFS), overall survival (OS), safety, and pharmacokinetics. Patients received the recommended phase 2 dose (1 mg/day; 0.75 mg/day moderate renal impairment).

**Results:**

Nineteen Japanese patients with g*BRCA1/2*-mutated locally advanced or metastatic breast cancer were enrolled. Confirmed ORR was 57.9% (11/19; 90% confidence interval [CI] 36.8–77.0). Stable disease was observed in 36.8% (7/19) of patients. Per investigator assessment, median PFS was 7.2 months (95% CI 4.1–not estimable) and 12-month OS rate was 84.7% (90% CI 57.5–95.1). Median OS was not reached; 17/19 patients were alive and censored at 12 months. All patients experienced treatment-related adverse events (AEs); the majority were hematologic. The most common treatment-related AE was anemia (68.4%; [13/19]). Grade 3/4 treatment-related AEs were observed in 52.6% (10/19) of patients. During the safety period, there were no grade 5 treatment-emergent AEs, treatment-related serious AEs, or deaths.

**Conclusions:**

In Japanese patients with g*BRCA* mutations and locally advanced or metastatic breast cancer, talazoparib monotherapy was generally well tolerated and resulted in clinically meaningful ORRs.

**Clinicaltrials.gov identifier:**

NCT03343054.

**Supplementary Information:**

The online version contains supplementary material available at 10.1007/s12282-022-01390-w.

## Introduction

Breast cancer remains the most frequently diagnosed cancer worldwide. In 2020, 2.26 million new cases were diagnosed and 685,000 deaths were attributed to breast cancer, including 92,000 new cases and 17,000 deaths in Japan [1], underscoring the significant unmet need for effective breast cancer treatments. Breast cancer is a heterogeneous disease, and treatment options and prognosis vary based on the hormone-receptor status and genetic profile of the individual patient [2].

The genome is susceptible to DNA damage, requiring DNA repair mechanisms to maintain genomic integrity [3]. Functional deficits in genes responsible for DNA damage repair (DDR) involved directly or indirectly in homologous repair recombination (HRR), such as *BRCA1* and *BRCA2*, are associated with an increased risk for multiple types of cancer [4, 5]. Germline defects in *BRCA1/2* can be found in approximately 10% of breast cancers [3]. Individuals with germline *BRCA1* mutations have a 57‒72% lifetime probability of developing breast cancer, whereas those with germline *BRCA2* mutations have a 45‒69% lifetime probability [6–8]. Among women with human epidermal growth factor receptor 2 (HER2)-negative breast cancer, those with germline *BRCA1/2* mutations tend to be diagnosed at a younger age and more frequently have a family history of the disease [3]. Breast cancer develops earlier and generally has a more aggressive clinical course in women with germline *BRCA* mutations than those with somatic mutations [8, 9].

*BRCA1/2* genes are involved in HRR, which is a key mechanism in the repair of double-stranded DNA breaks [3, 4]. HRR-deficient cells become dependent on alternative methods of DNA repair, such as single-strand break repair [4], which is mediated by poly(ADP-ribose) polymerase (PARP). Talazoparib inhibits PARP1 and PARP2, two key enzymes involved in DDR, and effectively traps PARP on single-stranded DNA breaks, causing an accumulation of double-stranded DNA breaks that cannot be effectively repaired in cancer cells with mutations in DDR/HRR genes, including *BRCA1/2* [10–15]. Talazoparib is a PARP inhibitor that is approved as monotherapy for the treatment of patients with HER2-negative advanced breast cancer with a germline *BRCA1/2* mutation in the United States, United Kingdom, European Union, and other countries [16, 17].

In the phase 3 EMBRACA trial involving patients with advanced breast cancer and a germline *BRCA1/2* mutation, talazoparib monotherapy (oral, 1 mg once daily [QD]) demonstrated significantly longer median progression-free survival compared with standard chemotherapy (8.6 months vs 5.6 months; hazard ratio for disease progression or death, 0.54; 95% confidence interval [CI] 0.41–0.71; *P* < 0.001) [18]. However, there was no clinical experience with talazoparib in Japanese patients at the initiation of the EMBRACA trial. The half-maximal inhibitory concentration of talazoparib (4‒11 nM) is comparable to that of other PARP inhibitors that are approved or under investigation in Japan [19, 20], but talazoparib is far more effective at trapping PARP on DNA [19].

Recently, a phase 1 trial was undertaken to evaluate the safety, pharmacokinetic profile, and preliminary efficacy of talazoparib monotherapy in nine Japanese patients with advanced solid tumors, regardless of germline and/or somatic mutation status in DDR/HRR-related genes [21]. This study consisted of two parts: part 1 was a dose-escalation study and part 2 was a dose-expansion study. In the recently reported results from part 1, no dose-limiting toxicities were observed, and the majority of treatment-emergent adverse events were mild and/or moderate (grade ≤ 2) [21]. Talazoparib monotherapy was well tolerated and showed antitumor activity, with an overall disease control rate of 44.4%, including two patients with stable disease [21].

Here, we report the primary results of part 2 of the study, dose expansion in 19 Japanese patients with germline *BRCA1/2* mutations, and locally advanced or metastatic breast cancer.

## Methods

### Study design

This phase 1, open-label, multicenter study evaluated talazoparib monotherapy in Japanese patients with germline *BRCA* mutations and HER2-negative locally advanced or metastatic breast cancer (ClinicalTrials.gov NCT03343054). This study comprised two parts: a dose-escalation part in patients with solid tumors and a dose-expansion part in patients with advanced breast cancer. A schematic of the study design is provided in Online Resource 1. Previously reported results from the dose-escalation part established the recommended dose as 1 mg QD for the dose-expansion part of this study [21]. The objective of the dose-expansion part was to evaluate the efficacy, safety, and pharmacokinetics of talazoparib monotherapy in Japanese patients with germline *BRCA* mutations and HER2-negative locally advanced or metastatic breast cancer. The primary completion date for the dose-expansion part was January 11, 2021.

### Patients and treatment

For inclusion, all patients (female or male, aged ≥ 20 years) were required to have an Eastern Cooperative Oncology Group (ECOG) performance status ≤ 2, adequate organ function, histologically or cytologically confirmed carcinoma of the breast, and germline *BRCA* mutations. Key inclusion and exclusion criteria are listed in Online Resource 2.

Eligible patients received talazoparib at the recommended dose (1 mg QD) from Day 1 for each 28-day cycle. In patients with moderate renal impairment (creatinine clearance 30–59 mL/min), the starting dose was reduced by one dose level (0.25 mg/day) to 0.75 QD. In response to grade 1/2 treatment-related toxicities, no specific dose modifications were recommended, except for patients with moderate renal impairment, in which case the starting dose was reduced by one dose level. For grade ≥ 3 events of anemia (hemoglobin < 8.0 g/dL), neutropenia (ANC < 1000/μL), or thrombocytopenia (platelets < 50,000/μL), supportive care was administered and treatment was interrupted until the event resolved. For neutropenia and thrombocytopenia, if the event resolved in 1 week or less, talazoparib was resumed at the same dosage. If the event resolved after a week, then talazoparib was resumed at the next lower dosage level. For anemia, dosing was resumed at the next lowest dose level if the event lasted less than 4 weeks. Talazoparib was discontinued for all anemia, neutropenia, and thrombocytopenia grade ≥ 3 events lasting longer than 4 weeks.

### Endpoints

The primary endpoint of the dose-expansion part was confirmed objective response rate determined by investigator assessment (Response Evaluation Criteria in Solid Tumors 1.1). Key secondary efficacy endpoints included objective response rate by blinded independent central review assessment; disease control rate at 16 and 24 weeks defined as patients with confirmed complete response, confirmed partial response, and/or stable disease; and time-to-tumor response, duration of response, progression-free survival, and overall survival. Secondary safety endpoints included type, frequency, and severity of adverse events (as graded by the National Cancer Institute-Common Terminology Criteria for Adverse Events [NCI-CTCAE], version 4.03), and laboratory abnormalities.

To evaluate the pharmacokinetics of talazoparib monotherapy, talazoparib trough concentrations (*C*_trough_) were evaluated at Day 1 of Cycle 2, 3, and 4 as a secondary endpoint. *C*_trough_ concentrations at steady state were analyzed descriptively by cycle and day. *C*_trough_ concentration at steady state is defined as the pre-dose concentration that meets the following dose-compliant acceptance criteria: the patient must have received the same dose of talazoparib QD for 7 consecutive days before the pre-dose pharmacokinetic sampling, and pharmacokinetic samples must have been collected 24 h ± 10% after the dose administered the day before the pre-dose pharmacokinetic sampling.

### Statistical analysis

Assuming a confirmed talazoparib objective response rate of 50% and a null proportion of 18.4% based on the results of EMBRACA [18], 17 patients are needed to preserve an 80% probability of the lower limit of the two-sided 90% CI of the confirmed objective response rate exceeding the null proportion of 18.4%. If the lower limit of the two-sided 90% CI of confirmed objective response rate exceeds 18.4%, it is considered that talazoparib shows clinically meaningful antitumor activity in Japanese patients with germline *BRCA* mutations and locally advanced or metastatic breast cancer.

All analyses for primary and secondary endpoints based on tumor burden (i.e., objective response rate, disease control rate, duration of response, or progression-free survival) were performed by investigator assessment and independent radiology assessment. For analysis of the primary endpoint, the number, percent, and exact two-sided 90% CI were calculated. Analyses of the other binary secondary endpoints (objective response rate assessed by blinded independent central review and disease control rate) were performed using the same methods as the primary analysis. Time-to-event endpoints (time-to-tumor response, progression-free survival, duration of response, and overall survival) were descriptively summarized using the Kaplan–Meier method.

## Results

### Patients and disposition

In this study, a total of 79 patients were prescreened for *BRCA* mutations using BRACAnalysis CDx^™^ (Online Resource 3). Deleterious or suspected deleterious germline *BRCA1* or *BRCA2* mutations were detected in seven patients. The other screened patients were passed through prescreening based on historical results generated previously using BRACAnalysis CDx^™^. In total, 22 germline *BRCA*-positive patients were identified through screening: three failed screening and were not enrolled, and 19 patients were enrolled in the expansion cohort, all of whom were treated. Reasons for screening failure were lack of deleterious or suspected deleterious germline *BRCA1* or* 2* mutation*,* inadequate organ function, and metastases in the central nervous system or leptomeningeal carcinomatosis. The 19 patients in the expansion cohort composed both the Safety Analysis Set and the Full Analysis Set.

The mean (range) age of patients in the dose-expansion part was 54.5 years (32‒77). Most (89.5%; 17/19) patients had an ECOG performance status of 0, while 10.5% (2/19) had an ECOG performance status of 1 (Table 1). Disease characteristics are summarized in Table 1. Ten patients (52.6%) had hormone-receptor-positive (HR+) breast cancer and nine (47.4%) had triple-negative breast cancer (TNBC). *BRCA2* mutations (73.7%; 14/19) were more common than *BRCA1* (26.3%; 5/19) mutations. Of the 14 patients with *BRCA2* mutations, five had TNBC and nine had HR+ disease. Of the five patients with *BRCA1* mutations, four had TNBC and one had HR+ breast cancer.

**Table 1:** Baseline patient and disease characteristics

	Talazoparib (*N* = 19)
Age (years), *n* (%)	
18–44	7 (36.8)
45–64	6 (31.6)
≥ 65	6 (31.6)
Mean	54.5
Range	(32–77)
Race, *n* (%)	
Japanese	19 (100.0)
ECOG performance status, *n* (%)	
0	17 (89.5)
1	2 (10.5)
2	0
Primary diagnosis, *n* (%)	
HR+/HER2 negative breast cancer	10 (52.6)
TNBC	9 (47.4)
*BRCA* mutational status, *n* (%)	
*BRCA1*	5 (26.3)
*BRCA2*	14 (73.7)
Number of prior systemic medications for advanced or metastatic breast cancer, *n* (%)	
0 regimens	9 (47.4)
1 regimen	7 (36.8)
2 regimens	0
3 regimens	2 (10.5)
≥ 4 regimens	1 (5.3)
Number of prior adjuvant and neo-adjuvant medications, *n* (%)	
0 regimens	4 (21.1)
1 regimen	1 (5.3)
2 regimens	6 (31.6)
3 regimens	6 (31.6)
≥ 4 regimens	2 (10.5)

The denominator to calculate percentages is *N*, the number of patients in the Full Analysis Set within each treatment group

*ECOG* Eastern Cooperative Oncology Group, *HER2* human epidermal growth factor receptor 2, *HR*+ hormone-receptor-positive, *TNBC* triple-negative breast cancer

Regarding prior systemic therapies for advanced breast cancer, including the adjuvant setting, 94.7% (18/19) of patients received anthracycline and/or taxane-based therapies, 21.1% (4/19) were treated with cyclin-dependent kinase inhibitors (abemaciclib or palbociclib), and 5.3% (1/19) were treated with a platinum-based regimen as adjuvant therapy. Additional information about prior treatments (prior systemic therapy, prior radiation, and prior surgery) is provided in Online Resource 4.

### Efficacy

**Fig. 1:**
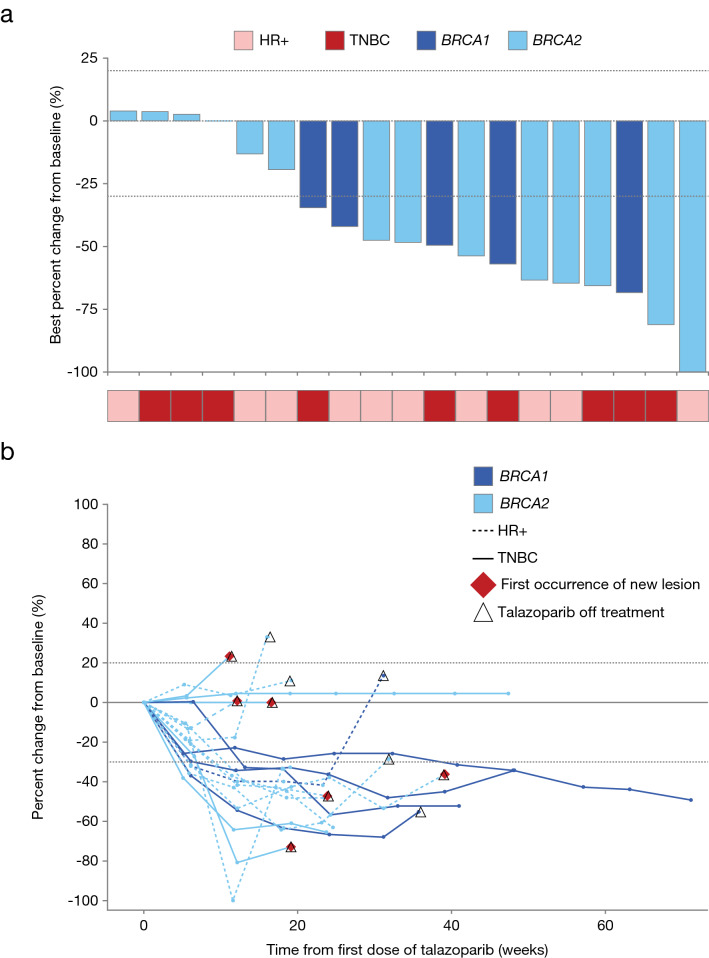
Best percent change in sum of diameters for target lesions per investigator assessment (RECIST 1.1) for each patient. **a** Percent change from baseline in the sum of diameters for target lesions. Only includes patients with target lesions at baseline and at least one non-missing post-baseline percent change from baseline assessment up to time of progressive disease or new anti-cancer therapy. The fourth patient from the left in panel A is *BRCA2*-positive. **b** Time course of percent change in sum of diameters for target lesion. Only includes patients with target lesions at baseline and at least one adequate post-baseline assessment. *HR+* hormone-receptor-positive, *RECIST* Response Evaluation Criteria in Solid Tumors, *TNBC* triple-negative breast cancer

The primary endpoint was objective response rate based on investigator assessment. To be considered clinically meaningful, the lower limit of the 90% CI of the confirmed objective responses, defined as complete and partial responses, needed to exceed the null proportion of 18.4%. Confirmed objective responses to talazoparib were observed in 57.9% (11/19; 90% CI 36.8–77.0) of patients, indicating clinically meaningful antitumor activity, although no patients experienced a complete response. Stable disease was observed in 36.8% (7/19) of patients, and 5.3% (1/19) showed progressive disease. A disease control rate up to week 16 of 94.7% (18/19; 90% CI 77.4–99.7) was achieved as determined by confirmed best overall response based on investigator assessment and was stable up to week 24. A similar trend was observed when evaluated by blinded independent central review, which showed a confirmed objective response rate of 52.6% (10/19; 90% CI 32.0–72.6), and a disease control rate up to week 16 of 89.5% (17/19, 90% CI 70.4–98.1), which was also stable up to week 24. During the dose-expansion part, 78.9% (15/19) of the patients had at least some degree of tumor shrinkage in their target lesions. Figure 1a shows the percent change from baseline in the size of target lesions, and Fig. 1b shows the time course of percent change in target lesion size.

**Fig. 2:**
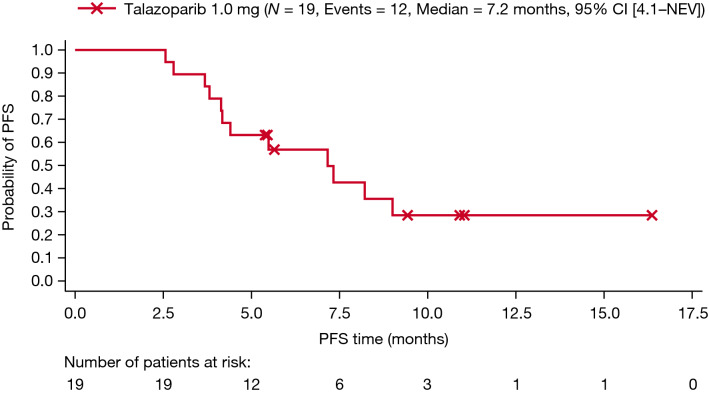
Kaplan–Meier plot of PFS assessed by investigator assessment per RECIST 1.1. One month is equivalent to 30.4375 days. *CI* confidence interval, *NEV* not evaluable, *PFS* progression-free survival, *RECIST* Response Evaluation Criteria in Solid Tumors

**Fig. 3:**
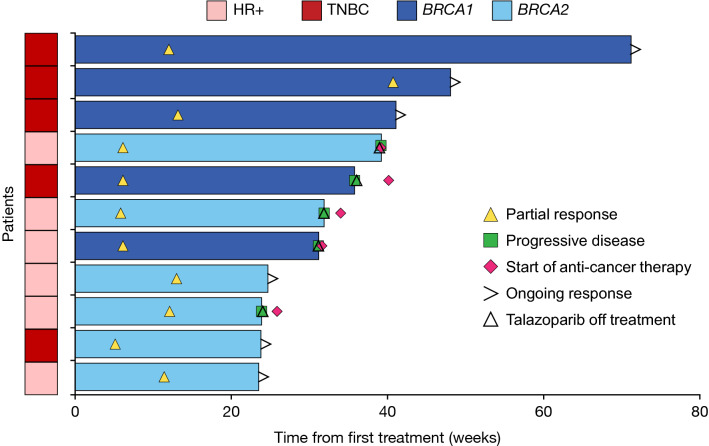
Time to and duration of response for the 11 patients with a confirmed response. *HR*+ hormone-receptor-positive, *TNBC* triple-negative breast cancer

Among the 11 patients with confirmed responses, the time to response ranged from 1.2 to 9.4 months, with a mean (standard deviation [SD]) time to response of 2.8 (2.3) months. Five patients (5/11; 45.5%) with confirmed responses experienced progressive disease during the study, while 54.5% (6/11) did not progress. The follow-up period for each patient was different. Based on Kaplan–Meier analysis, the probability of remaining progression-free was 0.57 (95% CI 0.32–0.76) at 6 months and 0.28 (95% CI 0.09–0.51) at 12 months. The median progression-free survival was 7.2 months (95% CI 4.1–not estimable [NE]) per investigator assessment, as shown in Fig. 2. The median Kaplan–Meier estimate of the duration of response was 6.8 months (95% CI 2.7–NE). The time to response and duration of response for each patient are illustrated in Fig. 3.

The cut-off date was 24 weeks (6 months) after the first study dose of the last enrolled patient. As of the data cut-off, there were two (2/19; 10.5%) deaths and the other 17 surviving patients (17/19; 89.5%) were censored. The overall survival rate at 12 months was 84.7% (90% CI 57.5–95.1). Median overall survival was not reached, as 17/19 patients were alive and censored at 12 months (Online Resource 5). In the following period, ten patients (52.6%) received anti-cancer therapies, two (10.5%) received radiation therapy, and one (5.3%) received surgery (Online Resource 6).

### Safety

An overall summary of adverse events is shown in Table 2. All 19 patients experienced treatment-related adverse events. Grade 3 or 4 treatment-related adverse events were observed in 52.6% (10/19) of patients and no grade 5 treatment-emergent adverse events were reported. Adverse events led to talazoparib interruption for 42.1% (8/19) of patients and included anemia (31.6%; [6/19]), decreased neutrophil count (10.5%; [2/19]), dyspnea (5.3%; [1/19]), and headache (5.3%; [1/19]). The most common (≥ 10% of patients) treatment-related adverse events are listed in Table 3. The majority of adverse events were hematologic in nature and the most common hematologic adverse event was anemia, which occurred in 68.4% (13/19) of patients. Grade 3 anemia was observed in 47.4% (9/19) of patients; there were no instances of grade ≥ 4 anemia. Neutropenia was observed in 63.2% (12/19) of patients, of which four experienced grade 3 neutropenia and there were no occurrences of grade ≥ 4.

**Table 2:** Overall summary of treatment-related adverse events

	Talazoparib (*N* = 19)
Patients evaluable for AEs, *n* (%)	19 (100)
Any AEs, *n* (%)	19 (100)
Serious AEs, *n* (%)	0
Maximum grade 3 or 4 AEs, *n* (%)	10 (52.6)
Maximum grade 5 AEs, *n* (%)	0
Discontinuations from study due to AEs^a^, *n* (%)	0
Study-drug discontinuations due to AE and continue study^b^, *n* (%)	0
Study-drug interruption due to AEs, *n* (%)	8 (42.1)
Dose reduction due to AEs, *n* (%)	9 (47.4)

Includes data up to 30 days after last dose of study drug. Except for the number of AEs, patients are counted only once per treatment in each row. AEs graded by the NCI-CTCAE, Version 4.03. Serious AEs are according to the investigator’s assessment

*AE* adverse event, *NCI-CTCAE* National Cancer Institute-Common Terminology Criteria for Adverse Events

^a^Patients who have an AE record that indicates the AE caused the patient to be discontinued from the study

^b^Patients who have an AE record that indicates the action taken with study treatment was drug withdrawn, but the AE did not cause the patient to be discontinued from the study

**Table 3:** Treatment-related adverse events occurring in ≥ 10% of patients

	Talazoparib (*N* = 19)
Number of patients by preferred term, *n* (%)	
ANEMIA^a^	13 (68.4)
Grade ≥ 3	9 (47.4)
NEUTROPENIA^a^	12 (63.2)
Grade ≥ 3	4 (21.1)
LEUKOPENIA^a^	8 (42.1)
Grade ≥ 3	2 (10.5)
Alopecia^a,b^	6 (31.6)
Stomatitis^a,b^	6 (31.6)
THROMBOCYTOPENIA^a,b^	6 (31.6)
Malaise^a,b^	5 (26.3)
Constipation^a,b^	3 (15.8)
Dizziness^a,b^	3 (15.8)
Headache^a,b^	3 (15.8)
Nausea^a,b^	3 (15.8)
Dysgeusia^a,b^	2 (10.5)
Dyspnea^a,b^	2 (10.5)
Fatigue^a,b^	2 (10.5)

Patients are only counted once per treatment per event. MedDRA v23.1 coding dictionary applied. AEs graded by the NCI-CTCAE, Version 4.03. Includes data up to 30 days after last dose of study drug

The following preferred terms fall under each cluster term: *ANEMIA* Anemia or Hematocrit decreased or Hemoglobin decreased, *LEUKOPENIA* Leukopenia or White blood cell count decreased, *LYMPHOPENIA* Lymphocyte count decreased or Lymphopenia, *NEUTROPENIA* Neutropenia or Neutrophil count decreased, *THROMBOCYTOPENIA* Platelet count decreased or Thrombocytopenia

*AE* adverse event, *MedDRA* Medical Dictionary for Regulatory Activities, *NCI-CTCAE* National Cancer Institute-Common Terminology Criteria for Adverse Events

^a^All grades

^b^No ≥ 3 events occurred

**Fig. 4:**
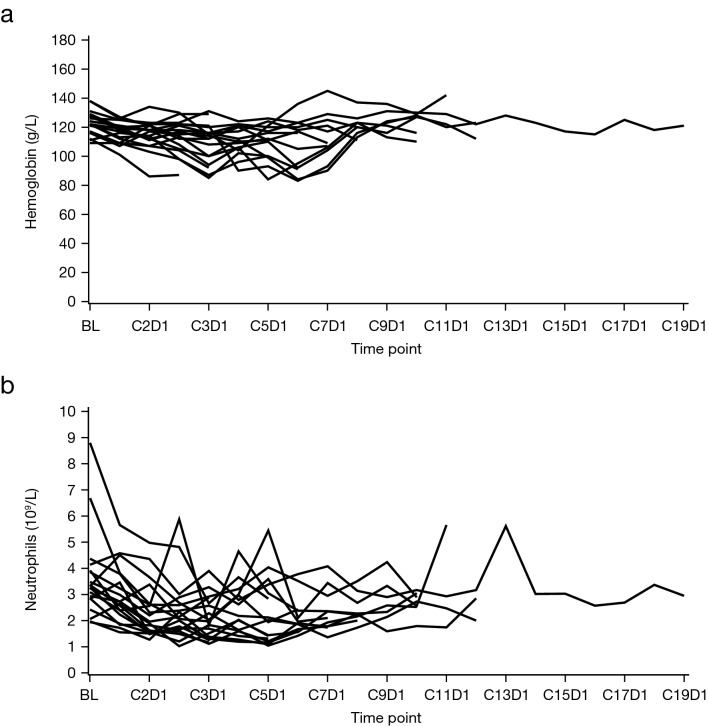
Individual results by visit for **a** hemoglobin level and **b** neutrophil count. Study baseline value is defined as the last value collected on or prior to the first dose date of study drug. *BL* baseline, *C* cycle, *D* day, *g* gram, *L* liter

The greatest mean decreases from baseline hemoglobin levels were observed at the beginning of Cycles 3 and 6 (mean change from baseline [SD] –14.1 [11.6] g/L and –16.7 [15.4] g/L, respectively) (Fig. 4a). Four patients (4/19; 21.1%) required red blood cell transfusions between Days 92 and 106 of treatment. The greatest mean decrease in neutrophil count compared with baseline was observed at Cycle 3 Day 1 (mean change from baseline [SD] –1.6 [1.6] ANC × 10^9^/L) (Fig. 4b). The median (range) time to the first onset of grade 3 anemia and neutropenia was 85 (71–176) days and 57 (29–106) days, respectively.

Dose reductions were implemented for 47.4% (9/19) of patients due to adverse events, including anemia (42.1%; [8/19]), decreased neutrophil count (21.1%; [4/19]), and decreased platelet count (5.3%; [1/19]). No patients discontinued the study due to adverse events. There were no serious treatment-related adverse events (Table 2) or deaths during the study period. Two patients died during the follow-up period: one patient died 194 days after the first day of talazoparib therapy due to an unknown cause, and one patient died due to disease progression 345 days after the first day of talazoparib therapy. One serious adverse event of aggravated cholelithiasis occurred in a 49-year-old patient, which was not treatment-related. The event was resolved in 8 days and there was no interruption or alteration in the talazoparib dose.

### Pharmacokinetics

Following multiple doses of talazoparib, the geometric mean talazoparib *C*_trough_ was similar throughout Cycles 2 through 4. The *C*_trough_ values of talazoparib were 3098 pg/mL for Cycle 2 Day 1, 3423 pg/mL for Cycle 3 Day 1, and 2910 pg/mL for Cycle 4 Day 1. The geometric mean *C*_trough_ of within-patient trough values, which was calculated using steady-state trough concentrations at each visit for each patient, was determined to be 3346 pg/mL.

## Discussion

The first-in-human phase 1 study of talazoparib included 113 patients in the United States and United Kingdom with advanced malignancies who had germline *BRCA1/2* mutations or a strong preclinical rationale for receiving a PARP inhibitor. Analysis of the dose-escalation part of the study, which included 39 patients and evaluated the safety, pharmacokinetic, and pharmacodynamic of talazoparib monotherapy, indicated a recommended dose of 1 mg daily [22]. The EMBRACA trial expanded upon these results, but did not include Japanese women [18]. The current study, which consisted of two parts (dose-escalation and dose-expansion), was undertaken to evaluate the safety, pharmacokinetics, and preliminary efficacy of talazoparib in Japanese patients. The dose-escalation part of this study included nine Japanese patients with locally advanced or metastatic solid tumors, who were unselected for mutations in DDR/HRR-related genes. Talazoparib was generally well tolerated, preliminary antitumor activity was observed, and the recommended phase 2 dose of talazoparib was determined to be 1 mg QD in this patient population [21]. In this dose-expansion study in 19 Japanese women with germline *BRCA* mutations and locally advanced or metastatic breast cancer receiving continuous dosing with talazoparib monotherapy (1 mg QD), the primary endpoint, confirmed objective response rate, was 57.9% (11/19; 90% CI 36.8–77.0). The lower limit of the 90% CI (36.8%) exceeded the null proportion of 18.4%, indicating clinically meaningful antitumor activity. The unconfirmed objective response rate was 68.4% (90% CI 47.0–85.3) on investigator assessment, with a median progression-free survival of 7.2 months. Results observed in this population are comparable to those seen in non-Japanese patients. In the phase 3 EMBRACA trial, the unconfirmed response rate to talazoparib was 62.6% (95% CI 55.8–69.0) on investigator assessment, and median progression-free survival was 8.6 months among those treated with talazoparib [18].

All patients experienced treatment-related adverse events, with 42.1% limited to grade 1 or 2. All treatment-related adverse events grade ≥ 3 were hematologic in nature, anemia being the most common. Five participants required red blood cell transfusions for anemia. In the phase 3 EMBRACA trial, grade 3 or 4 hematologic adverse events were also common, observed in 55% of patients who received talazoparib [18]. In the EMBRACA trial, the median (range) time from first talazoparib dose to onset of first grade ≥ 3 episode of anemia and neutropenia was 83 (13–961) and 50 (1–947) days, respectively [23]. Here, we observed that the median (range) time to the first onset of grade 3 anemia and neutropenia was 85 (79‒176) days and 57 (29‒106) days, respectively.

The *C*_trough_ values were similar between Cycles 2 through 4, indicating no substantial change in talazoparib *C*_trough_ once steady-state levels were reached. The mean *C*_trough_, based on data from Day 1 of Cycles 2, 3, and 4, was determined to be 3.35 ng/mL, which is similar to observations from the part 1 dose-escalation in which the mean *C*_trough_ was 3.65 ng/mL for the six patients who received multiple 1.0 mg daily doses of talazoparib [21]. Additionally, in the EMBRACA trial, which did not enroll patients within Japan, the geometric mean *C*_trough_ at steady state was found to be 3.53 ng/mL [24]. Thus, the geometric mean *C*_trough_ within-patient values in this Japanese study were comparable to the result seen in non-Japanese patients.

Overall, this study demonstrated that talazoparib monotherapy (1 mg QD) resulted in clinically meaningful objective responses and was well tolerated in Japanese patients with germline *BRCA* mutations and locally advanced or metastatic breast cancer, in keeping with findings from trials that included non-Japanese patients.

## Supplementary Information

Below is the link to the electronic supplementary material.

Supplementary file1 (DOCX 276 KB)

## Data Availability

Upon request, and subject to review, Pfizer will provide the data that support the findings of this study. Subject to certain criteria, conditions and exceptions, Pfizer may also provide access to the related individual de-identified participant data. See https://www.pfizer.com/science/clinical-trials/trial-data-and-results for more information.
