# Supplementary material for: Efficacy and safety of talazoparib in Japanese patients with germline BRCA-mutated locally advanced or metastatic breast cancer: results of the phase 1 dose-expansion study
Source: Breast Cancer. 2022 Jul 30;29(6):1088–98. doi: 10.1007/s12282-022-01390-w (PMC9587945; doi:10.1007/s12282-022-01390-w)
Supplement: Supplementary file 1 — Supplementary file1 (DOCX 276 KB) [file 12282_2022_1390_MOESM1_ESM.docx]

***Breast Cancer***

**Supplementary materials**

**Efficacy and safety of talazoparib in Japanese patients with germline *BRCA-*mutated locally advanced or metastatic breast cancer: results of the phase 1 dose-expansion study**

Haruru Kotani, Norikazu Masuda, Toshinari Yamashita, Yoichi Naito, Tetsuhiko Taira, Kenichi Inoue, Masato Takahashi, Kan Yonemori, Shigeyuki Toyoizumi, Yuko Mori, Takashi Nagasawa, Natsuki Hori, Hiroji Iwata

**Corresponding author and contact details:**

Haruru Kotani, Aichi Cancer Center, 1-1 Kanokoden, Chikusa-ku, Nagoya 464-8681, Japan

**Email:** k.haruru@aichi-cc.jp
**Phone:** +81-52-762-6111

**Fax:** +81-52-764-2963

**Online Resource 1** Study design

**
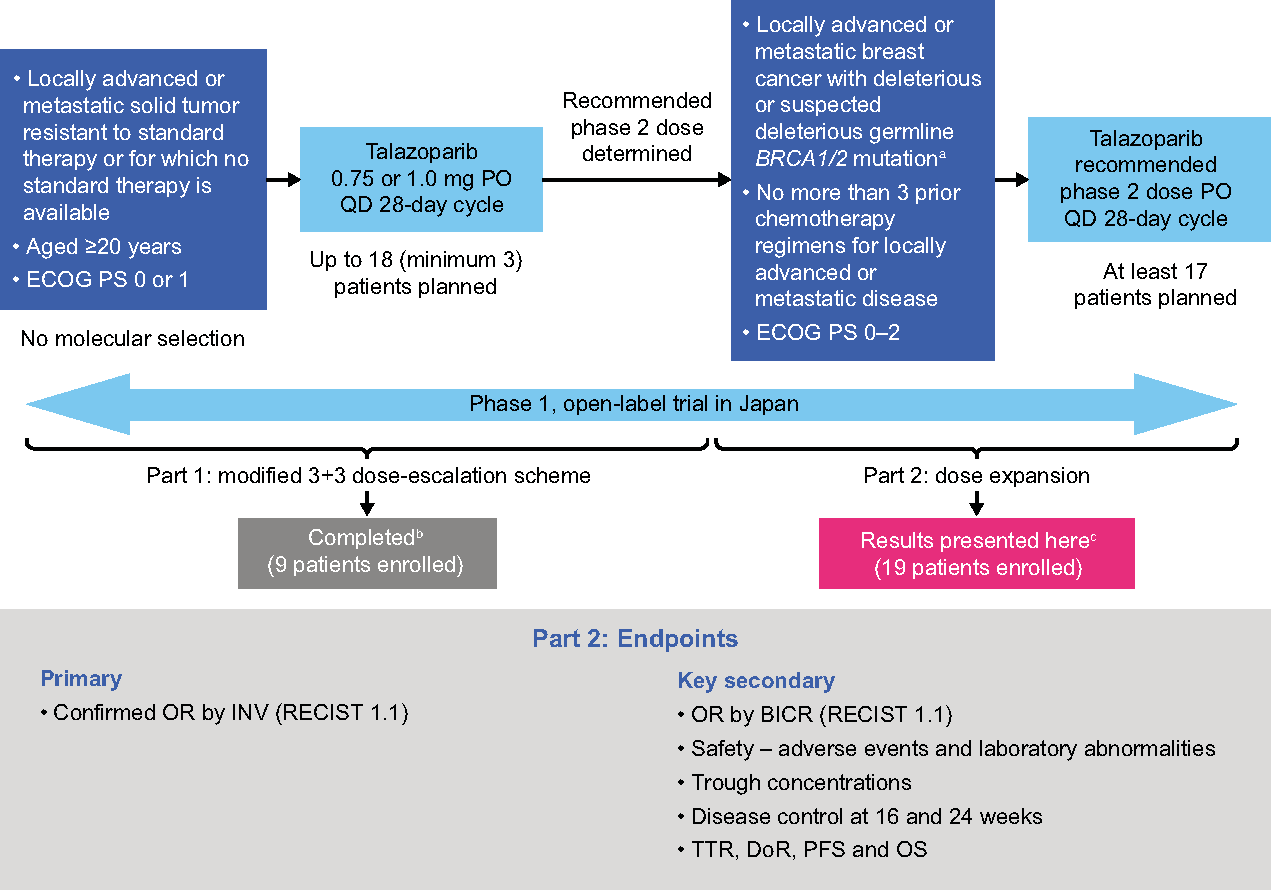
**

www.clinicaltrials.gov (NCT03343054)

*BICR* blinded independent central review assessment; *DoR* duration of response; *ECOG* Eastern Cooperative Oncology Group; *INV* investigator assessment; *OR* objective response; *OS* overall survival; *PFS* progression-free survival; *PO* orally; *PS* performance status; *QD* once daily; *RECIST* Response Evaluation Criteria in Solid Tumors; *TTR* time-to-tumor response
^a^Human epidermal growth factor receptor 2-positive breast cancer is excluded

^b^Data cutoff date: January 2020

^c^Data cutoff date: January 11, 2021

**Online Resource 2** Key inclusion and exclusion criteria

| **Inclusion criteria** |
| --- |
| - Histologically or cytologically confirmed carcinoma of the breast^a^ |
| - Locally advanced breast cancer that is not amenable to curative radiation or surgery and/or metastatic disease |
| - Deleterious, suspected deleterious, or pathogenic g*BRCA1/2* mutations^b^ |
| - ≤3 prior chemotherapy-inclusive regimens |
| - Prior treatment with a taxane and/or anthracycline in the neo-adjuvant, adjuvant, locally advanced, or metastatic setting, unless medically contraindicated |
| - Measurable lesion(s) by RECIST 1.1 |
| - Able to take oral medication |
| - ECOG performance status ≤2 |
| - Adequate organ function^c^ |
| **Exclusion criteria** |
| - No prior neo-adjuvant/adjuvant chemotherapy^d^ |
| - Prior treatment with a PARP inhibitor (not including iniparib) - Objective disease progression while receiving platinum chemotherapy administered for locally advanced or metastatic disease |
| - Prior treatment, within 14 days before starting study, with cytotoxic chemotherapy, radiation therapy, antihormonal therapy, or other targeted anti-cancer therapy |
| - HER2-positive breast cancer |
| - Active inflammatory breast cancer |
| - Central nervous system metastases^e^ |

*ALT* alanine aminotransferase; *AST;* aspartate aminotransferase; *dL* deciliter; *ECOG* Eastern Cooperative Oncology Group; *g* grams; *gBRACA1/2* germline *BRCA1* and/or *BRCA2;* *HER2* human epidermal growth factor receptor 2; *L* liter; *PARPi* poly(ADP-ribose) polymerase enzyme inhibitor; *mL* milliliter; *min* minute; *RECIST* Response Evaluation Criteria in Solid Tumors; *ULN* upper limit of normal

^a^Locally advanced breast cancer that was not amenable to curative radiation or surgery and/or metastatic disease

^b^Confirmed by BRACAnalysis CDx^TM^ (Myriad Genetics, Inc.)

^c^Defined by serum AST and ALT ≤2.5 × ULN; if liver function abnormalities are due to hepatic metastasis, then AST and ALT ≤5 × ULN, total serum bilirubin ≤1.5 × ULN (≤3 × ULN for Gilbert’s syndrome), alkaline phosphatase ≤2.5 × ULN (≤5 × ULN in case of bone metastasis), hemoglobin ≥9.0 g/dL with last transfusion at least 14 days prior to the first dose of study treatment, absolute neutrophil count ≥1.5 × 10^9^/L without the use of growth factor, platelet count ≥100 x 10^9^/L without the use of platelet transfusions or growth factor, and estimated creatinine clearance ≥30 mL/min

^d^Unless the investigator determined that treatment with a PARPi such as talazoparib would be in the best interest of the subject if they had received prior treatment with a PARPi (not including iniparib)

^e^Except adequately treated brain metastases documented by baseline CT or MRI scan that had not progressed since previous scans and that did not require corticosteroids (prednisone ≤5 mg/day or equivalent was allowed) for the management of central nervous system symptoms. Patients with leptomeningeal carcinomatosis are excluded

**Online Resource 3** Summary of *BRCA* testing (screened and full analysis set)

|  | **Number of patients tested at time of screening** | **Number of patients tested prior to screening** | **Total** |
| --- | --- | --- | --- |
| **Population: screened** | 79 | 15 | 94 |
| Negative | 72 | 0 | 72 |
| Positive | 7 | 15 | 22 |
| *BRCA1* | 2 | 4 | 6 |
| *BRCA2* | 5 | 11 | 16 |
|  |  |  |  |
| **Population: Full Analysis Set** | 5 | 14 | 19 |
| Negative | 0 | 0 | 0 |
| Positive | 5 | 14 | 19 |
| *BRCA1* | 2 | 3 | 5 |
| *BRCA2* | 3 | 11 | 14 |

**Online Resource 4** Summary of prior therapies for advanced breast cancer, prior radiation therapy, and prior surgery

|  | **Talazoparib 1.0 mg (*N* = 19)** |
| --- | --- |
| **Prior systemic therapies – any, *n (%)*** | 18 (94.7) |
| Cytotoxic therapies^a^ | 18 (94.7) |
| Hormonal therapies^b^ | 9 (47.4) |
| Kinase inhibitors^c^ | 4 (21.1) |
| Monoclonal antibodies^d^ | 7 (36.8) |
| Blinded therapy | 1 (5.3) |
| Investigational drug | 1 (5.3) |
| **Prior radiation therapy, *n (%)*** |  |
| Radiotherapy | 11 (57.9) |
| **Prior surgery, *n (%)*** | 15 (78.9) |
| Axillary lymphadenectomy | 3 (15.8) |
| Breast-conserving surgery | 4 (21.1) |
| Chest-wall resection | 1 (5.3) |
| Lymphadenectomy | 2 (10.5) |
| Mastectomy | 9 (47.4) |
| Modified radical mastectomy | 2 (10.5) |
| Nipple resection | 1 (5.3) |
| Simple mastectomy | 3 (15.8) |
| Thoracic operation | 1 (5.3) |
| Tumor excision | 1 (5.3) |

Patients are counted only once for a specific procedure in the table body

^a^Cytotoxic therapies included capecitabine; carboplatin; cyclophosphamide; cyclophosphamide monohydrate; docetaxel; doxorubicin; doxorubicin hydrochloride; epirubicin hydrochloride; fluorouracil; gemcitabine hydrochloride; paclitaxel; vinorelbine tartrate

^b^Hormonal therapies included anastrozole, exemestane, fulvestrant, goserelin acetate, letrozole, leuprorelin acetate, tamoxifen, and tamoxifen citrate

^c^Kinase inhibitors included abemaciclib, everolimus, and palbociclib

^d^Monoclonal antibodies included bevacizumab, denosumab, nivolumab, and trastuzumab

**Online Resource 5** Kaplan-Meier plot of overall survival

**
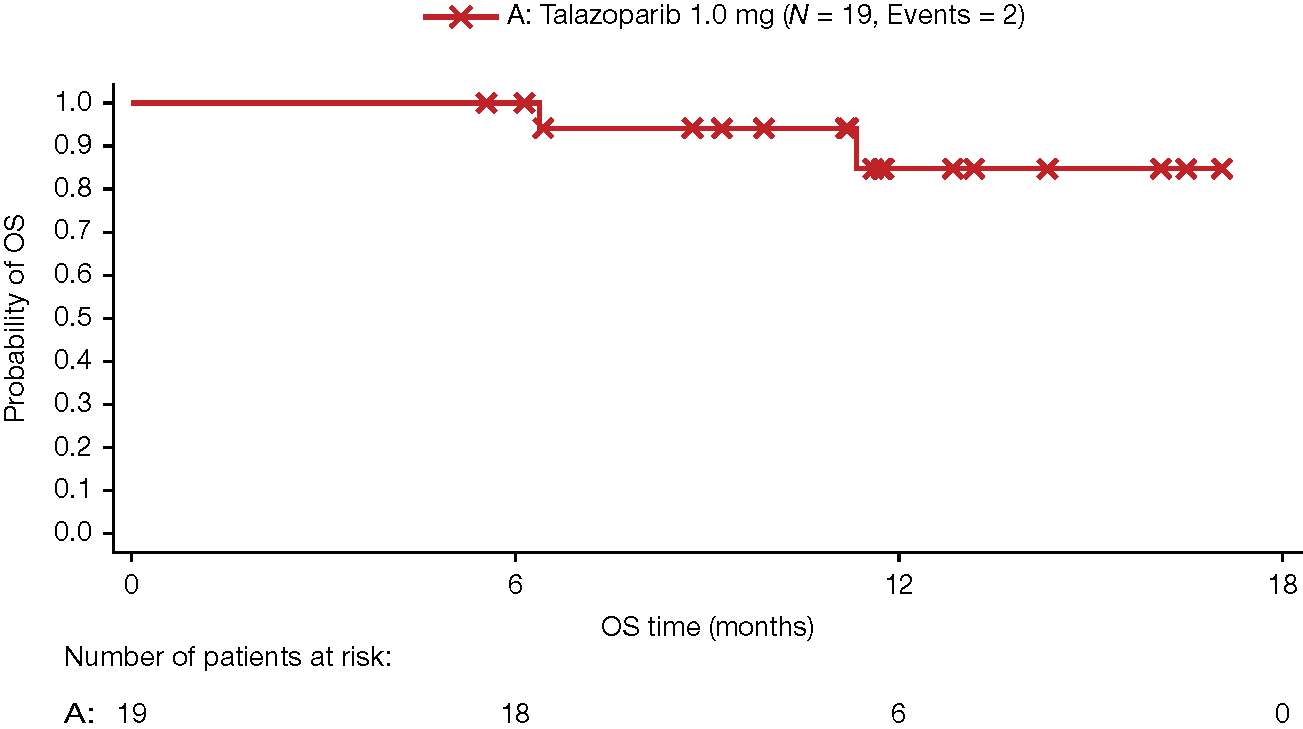
**

One month is equivalent to 30.4375 days

*OS* overall survival

**Online Resource 6** Summary of follow-up therapies

|  | **Talazoparib 1.0 mg**  **(*N* = 19)** |
| --- | --- |
| **Patients with any follow-up cancer therapies, *n (%)***^a^ | 10 (52.6) |
| Antiemetics and antinauseants^b^ | 1 (5.3) |
| Antineoplastic agents^c^ | 10 (52.6) |
| Cardiac therapy^d^ | 5 (26.3) |
| Endocrine therapy^e^ | 4 (21.1) |
| Investigational drug^f^ | 1 (5.3) |
| Ophthalmologicals^g^ | 4 (21.1) |
| **Patients with ≥1 follow-up radiation therapy, *n (%)***^h^ | 2 (10.5) |
| Radiotherapy | 2 (10.5) |
| **Patients with ≥1 follow-up surgeries, *n (%)***^h^ | 1 (5.3) |
| Breast-conserving surgery | 1 (5.3) |

*WHO DDE* World Health Organization Drug Dictionary Enhanced; *MedDRA* Medical Dictionary for Regulatory Activities

^a^WHO DDE v202003 coding dictionary applied

^b^Palonosetron hydrochloride

^c^Antineoplastic agents included abemaciclib, atezolizumab, bevacizumab, capecitabine, carboplatin, cyclophosphamide, docetaxel, eribulin mesilate, gemcitabine hydrochloride, gimeracil/oteracil potassium/ tegafur, paclitaxel, palbociclib, pertuzumab, trastuzumab, U3-1402

^d^Paclitaxel

^e^Endocrine therapies included anastrozole, fulvestrant, goserelin acetate, letrozole

^f^U3-1402

^g^Bevacizumab

^h^Patients are counted only once for a specific procedure. MedDRA v23.1 coding dictionary applied
